# Tooth loss in breast cancer patients: A comparison between tamoxifen-treated and non-treated patients

**DOI:** 10.4317/medoral.26528

**Published:** 2024-05-25

**Authors:** Jane Janet Julca-Baltazar, Angel Seven Asmat-Abanto, Andy Ray Pantoja-Lázaro, Ana Paula Gorritti-Rubio, Carlos Alberto Minchón-Medina

**Affiliations:** 1 Orcid: 0009-0007-3953-8296. Student of Stomatology Study Program. Antenor Orrego Private University, Trujillo, Peru; 2 Orcid: 0000-0001-5726-6692. Doctor in Stomatology. Specialist in Periodontics. Professor of Human Medicine Study Program. Antenor Orrego Private University, Trujillo, Peru; Professor of Stomatology Study Program. Antenor Orrego Private University, Trujillo, Peru; 3 Orcid: 0009-0008-4119-5832. Specialist in Oncological Surgery. Sub-specialist in Oncological Surgery of Breasts, Skin and Soft Tissues. Chief of the Department of Oncological Surgery of Breasts and Soft Tissue Tumors of the Regional Institute of Neoplastic Diseases IREN Norte “Dr. Luis Pinillos Ganoza”, Trujillo, Peru; 4 Orcid: 0009-0002-1486-9068. Specialist in Medical Oncology. Assistant physician of the Medical Oncology Service of the High Complexity Hospital "Virgen de la Puerta"; Professor of Human Medicine Study Program. Antenor Orrego Private University, Trujillo, Peru; 5 Orcid: 0000-0002-2441-5302. Doctor in Public Health, Postgraduate Professor. Department of Statistics, Trujillo National University, Trujillo, Peru

## Abstract

**Background:**

Tooth loss (TL) affects quality of life and general health. The literature suggesting that tamoxifen treatment in patients with breast cancer (BC) could be associated with alterations in oral health, increasing the risk of TL, is still scarce. This work aimed to determine the relationship between TL and tamoxifen consumption in patients with BC.

**Material and Methods:**

This cross-sectional observational study was carried out from July to September 2023 in the medical oncology services of the “Virgen de la Puerta” - ESSALUD High Complexity Hospital and “Dr. Luis Pinillos Ganoza” - IREN Norte - Regional Institute of Neoplastic Diseases, in Trujillo - Peru. Overall, 200 adult patients diagnosed with BC were evaluated, of which 100 consumed tamoxifen and 100 did not. Inter- and intra-rater reliability was determined with respect to TL, resulting in intra-class correlation values RHO = 0.971 and interclass RHO = 0.938. The oncologist of the corresponding service performed BC diagnosis and stage. Poisson regression was used to analyze results with a significance level of *p*<0.05.

**Results:**

No relationship was found between TL and tamoxifen consumption in patients with breast cancer (*p*= 0.221); however, greater TL was observed in women who consumed tamoxifen for more than one year compared to those who did not use it (*p*=0.025) and in older adult women compared to young women (*p*=0.030).

**Conclusions:**

There is a relationship between TL and time of use of tamoxifen in patients with BC, concluding that patients who consumed tamoxifen for more than one year had greater TL than those who did not. Furthermore, no relationship was found between TL and cancer stages, but there was greater TL in older adult patients and also in those who consumed tamoxifen and did not receive chemotherapy or radiotherapy.

** Key words:**Tooth loss, tamoxifen, breast cancer, chemotherapy, radiotherapy.

## Introduction

Dental caries (DC), periodontal disease (PD) and tooth loss (TL) are considered important public health problems (1). TL negatively affects the quality of life and general health of those who suffer from it (2). Recent studies indicate that 1 in 3 patients aged 65 years or older have lost 6 or more teeth and approximately 10% have lost all their teeth (3).

TL is associated with age, alcohol consumption, smoking, socioeconomic status and malnutrition (1,4). The absence of teeth causes the inability to chew correctly, which causes the patient to be more food selective, affecting correct nutrition and negatively impacting general health (5).

Although TL can be prevented with good oral hygiene and regular visits to the dentist (2,3), sometimes it cannot be easily controlled because some chronic diseases increase their probability of occurrence (3).

Cancer is a chronic disease characterized by abnormal and uncontrolled cell growth with poor immune system response (6). It is characterized by a wide spectrum of biological aggressiveness that impairs its adequate control (7,8).

Breast cancer (BC) is the second most common form of cancer in the world and the most prevalent among women, resulting in the fifth cause of death due to cancer and the first among women (1,9-14). However, in recent years, the incidence of BC has been reduced, probably due to early detection, adjuvant therapies and hormone therapy (12).

The choice of treatment for BC is based on factors such as tumor type, cancer stage, and patient’s prognosis (1). Tamoxifen is the first-line medication for the treatment and prevention of recurrence of hormone receptor-positive breast tumors (1,10,15). This medication aims to reduce the effect of estrogens on breast tissue (12), by blocking its receptors (16).

Despite its multiple benefits, tamoxifen has side effects such as vaginal dryness, endometrial cancer, hot flashes, sleep disorders, fatigue, thromboembolism, thrombocytopenia, leukopenia, and resistance to the same drug (17,18). Moreover, this drug can have an impact on general oral health (13), such as generating changes in the flora due to its antibacterial effect (19) or reducing the trabecular bone density, promoting some oral pathologies such as periodontal disease and consequent TL (20). In addition, the presence of xerostomia and gingival bleeding during and after treatment is quite common (13).

Currently, insufficient studies evaluate the possible oral cavity changes associated with tamoxifen consumption in patients with BC. For this reason, considering that TL affects the quality of life and general health of cancer patients, this work aimed to determine the relationship between TL and tamoxifen consumption in patients with BC, as well as associated factors, to provide relevant and preliminary knowledge for continuous improvement in clinical care guidelines, which will positively impact the quality of life of these patients.

## Material and Methods

The present study has a cross-sectional observational design and was developed at the “Virgen de la Puerta” High Complexity Hospital - EsSalud and at the “Dr. Luis Pinillos Ganoza” - IREN Norte Regional Institute of Neoplastic Diseases (Trujillo, Peru), between July and September 2023.

The sample, selected using the accidental non-probabilistic method, was composed of 200 patients with breast cancer. This sample was calculated using the formula to compare the means of independent groups using data generated through a pilot study with 40 patients, with the following parameters: Zα=1.645 (Normal value with α=5% type I error), Zβ=0.842 (Normal value with β=20% type II error or 80% power), ₁=2.76 (Mean tooth loss in patients with BC who do not consume tamoxifen), ₂=1.62 (Mean tooth loss in patients with BC who consume tamoxifen), S2=10.47 (Tooth loss variance in patients with BC).

Patients included in the sample were over 18 years of age and attended outpatient consultations in the Medical Oncology service of the aforementioned hospitals. Pregnant patients, patients with modifying comorbidities or those that affect periodontal status, and those who did not agree to participate in the research were excluded.

To carry out this work, approval was obtained from the Faculty of Medicine (No. 2606-2023-FMEHU-UPAO), the ‘Antenor Orrego’ Private University Bioethics Committee (Bioethics Committee No. 0638- 2023-UPAO), the IREN Norte Research Bioethics Committee (CONSTANCIA No. 021-2023-IREN NORTE-CIEI), and the Training Directorate of the La Libertad Assistance Network - ESSALUD (PI No. 120 CIYE-O.C.I.Y. D-RALL-ESSALUD-2023). These operational units observed strict compliance with principles established in the Declaration of Helsinki of the World Medical Association and the General Health Law of Peru No. 26842.

Before participating, all patients received complete information about the research. They were given the informed consent form to read and sign if they agreed to participate. Subsequently, the main researcher examined the oral cavity of each patient lying on the stretcher of the oncology office with natural light and using oral mirrors, determining the missing teeth using the Decayed, Missing, and Filled Teeth Index (DMFT) without considering third molars; while the oncologist of the corresponding service determined BC diagnosis and stage. Data were recorded in the corresponding collection form, also recording basic demographic information and other covariates under study.

The reliability of the method for measuring DMFT was determined through intra- and inter-rater calibration of the main researcher and an expert specialist in Oral and Maxillofacial Surgery, with 27 years of hospital work, a Master's academic degree in Stomatology, and 17 years of university teaching practice. For this evaluation, 20 patients were examined, obtaining intra-rater intra-class correlations of 0.971 and inter-rater correlations of 0.938.

Data from the patients with breast cancer were incorporated into a database prepared in EXCEL and exported to Stata 16, with TL represented in Tables with means and standard deviations for each group, according to covariates. Prior to the statistical analysis, TL non-normality was tested using the Anderson-Darling test and Minitab 19. Given the non-normality and the fact that the number of teeth constitutes a discrete variable, the statistical analysis for the comparison of groups was performed using the Poisson regression model. Significance was considered if *p*<0.05.

## Results

In the present study, 200 women aged 20-75 years (X̅= 48.31; σ=9,935) with a diagnosis of breast cancer who attended at the “Virgen de la Puerta” High Complexity Hospital Oncology service and the Department of Breast and Soft Tissue Surgery of the “Dr. Luis Pinillos Ganoza” - IREN Norte Regional Institute of Neoplastic Diseases were evaluated between July and September 2023. The following results were obtained:

As can be observed in Table 1, the average TL was greater in the group that consumed tamoxifen (X̅ = 2.04) and even greater in the group that consumed it for more than one year (X̅ = 2.32); the average TL was greater in older patients (X̅ = 2.96), in cancer stages I (X̅ = 2.17) and IV (X̅ = 2.25) and in patients who received both chemotherapy and radiotherapy (X̅ = 2.54).

There was no relationship between TL and tamoxifen consumption, as shown in Table 2 (*p*=0.221). In addition, it was observed that women who used tamoxifen for ≤1 year (*p*=0.499) did not present greater TL. However, women who used tamoxifen for more than one year (*p*=0.025) presented greater TL than those who did not use the drug.

The information was expanded by evaluating TL in relation to the time of tamoxifen consumption in patients with breast cancer, adjusted for covariates:

In Table 3, taking as reference patients in stage I, no difference was found in TL compared to patients in stages II (*p*=0.383), III (*p*=0.574), and IV (*p*=0.547), but in older adult women (*p*=0.030) compared to young women, without difference between them and adults (*p*=0.085). Likewise, considering the interactive effects of tamoxifen consumption with the systemic cancer treatment received, in comparison with patients who did not use tamoxifen and did not receive any treatment, it was found that those who used tamoxifen and did not receive any treatment had greater TL (*p*=0.000) and those who did not use tamoxifen but received chemotherapy (*p*=0.008), radiotherapy (*p*=0.012) or both treatments (*p*=0.000). However, those who used tamoxifen and received chemotherapy (*p*=0.000), radiotherapy (*p*=0.005), or both treatments (*p*=0.000) presented less TL.

As shown in Table 4, no difference was found in the TL of patients with breast cancer in stages II-IV compared to those in stage I, and greater TL in older adult patients was also confirmed (*p*=0.034) compared to young patients. Regarding women who did not use tamoxifen and did not receive any systemic cancer treatment, greater TL was found in patients who used tamoxifen for less than one year (*p*=0.003) or more than one year (*p*=0.000) without systemic treatment, as well as those who did not use it but received chemotherapy (*p*=0.008), radiotherapy (*p*=0.012) or both (*p*=0.000). However, less TL was found in patients who used tamoxifen (regardless of duration) and received any treatments, except those who received tamoxifen for more than one year and radiotherapy (*p*=0.055).

## Discussion

Jardim *et al*. (13) indicate that treatment with tamoxifen affects the oral cavity in different ways. This drug is an estrogen modulator, which decreases bone density, causing alterations in oral health and consequently increasing the risk of TL (20).

The present investigation found that the average number of lost teeth is higher in patients who consume tamoxifen and even higher in patients who have been on treatment for more than one year. It was also evident that the average TL increased in people over 60 years of age, similar to results found by Araujo *et al*. (1), who indicated the risk of losing 12 teeth or more at older ages. This may occur because as a woman ages, the secretion of sex hormones decreases, thus increasing the risk of periodontitis (21). Furthermore, in this study, it was observed that the average TL is similar between the different cancer stages, probably because the stage was a covariate, requiring a larger sample size of each stage to obtain decisive conclusions. Concerning systemic cancer treatment, it was found that there is greater TL in patients who underwent both treatments (chemotherapy and radiotherapy), possibly because these therapies affect bone tissue, bacterial balance and salivary glands (22).

The present investigation aimed to determine the non-causal relationship between TL and tamoxifen consumption in patients with BC. The expected relationship was not found through bivariate analysis unless among patients who consumed tamoxifen for more than one year. This finding corroborates results found by Araujo *et al*. (1), probably because estrogen deficiency reduces salivary flow, decreasing the mouth pH and producing gingival inflammation, caries, and loss of attachment level. These authors also reported that BC patients treated with tamoxifen presented xerostomia, a burning sensation in the oral soft tissues, possibly due to alteration in oral homeostasis, which was associated with TL.

Since the number of patients per category was reduced, an attempt was made to adjust the entire sample according to covariates, applying the multivariate Poisson regression model to data, thus providing greater statistical power to the results. This analysis allowed improving the evaluation of the relationship between TL and tamoxifen consumption, eliminating the influence of confounding factors provided by covariates, finding a tendency towards higher TL in patients who consume tamoxifen, whether the consumption is less than one year or more than one year.

The multivariate model, in relation to TL, found data similar to the bivariate model concerning cancer stages and age; however, regarding systemic cancer treatment, it was found that patients who consumed tamoxifen and did not receive any other treatment, as well as those who did not use tamoxifen and underwent chemotherapy, radiotherapy or both, had greater TL. Likewise, the multivariate analysis of the use of tamoxifen in relation to the time of consumption corroborates the previous analysis; however, there is less TL in patients who consumed tamoxifen and received radiotherapy. Since systemic cancer treatment was a covariate in this study, subsequent studies should be carried out with an adequate sample size for each treatment group.

As a cross-sectional relationship study, the study of variables lacks temporal control, and in this sense, it does not allow for establishing a causal relationship, which is a limitation. However, this work offers interesting data that can serve as a basis for future analytical studies. It is necessary to carry out longitudinal investigations that take into account the moment of TL in relation to the start of medication with tamoxifen to know the potential effects of this drug on oral health.

The number of participants in this study was taken using the statistical methods described, which constitutes a noTable strength for the reliability of the findings. This allows an important contribution to the current literature on the topic. On the other hand, this work has clinical relevance since patients who receive oncological treatments perceive their oral health as compromised and may develop different alterations, such as periodontitis and TL. For this reason, dentists should be aware of these complications and implement preventive measures for these patients, effectively combining multidisciplinary care. Furthermore, oncologists should be aware of this possible relationship and integrate dental consultations into their clinical practice, taking into consideration that neglecting oral health would cause physical and psychological effects on patients. On the other hand, it is also important for patients to participate actively in their health care and request necessary dental appointments.

## Figures and Tables

**Table 1:** Tooth loss in patients with breast cancer according to age, cancer stage, systemic cancer treatment, and use of tamoxifen at the “Virgen de la Puerta” - EsSalud High Complexity Hospital and “Dr. Luis Pinillos Ganoza” - IREN Norte Regional Institute of Neoplastic Diseases (Trujillo, Peru), between July and September 2023.

Characteristics		Tooth loss		
		N	Mean	SD
Age	Young	3	0.33	0.58
	Adult	169	1.78	1.47
	Older adult	28	2.96	1.6
Cancer stage	I	36	2.17	1.48
	II	79	1.81	1.53
	III	81	1.9	1.58
	IV	4	2.25	2.06
Systemic cancer treatment	None	102	1.87	1.63
	Chemotherapy	56	1.73	1.38
	Radiotherapy	14	1.79	1.53
	Chemotherapy and radiotherapy	28	2.54	1.45
Use of tamoxifen	No	100	1.8	1.51
	Yes	100	2.04	1.58
Time of use of tamoxifen	No use	100	1.8	1.51
	Up to 1 year	41	1.63	1.77
	More than 1 year	59	2.32	1.37

**Table 2:** Tooth loss in relation to consumption and time of use of tamoxifen in patients with breast cancer.

Patients with breast cancer	Tooth loss	Coefficient	*p*
Use of tamoxifen	Yes	0.125	0.221
	Constant	0.588	0
Time of use of tamoxifen: Reference = no use	Up to 1 year	-0.097	0.499
	More than 1 year	0.255	0.025
	Constant	0.588	0

Poisson regression analysis.

**Table 3:** Tooth loss in relation to tamoxifen consumption in patients with breast cancer, adjusted for covariates.

Tooth loss		Coefficient	*p*
Use of tamoxifen: Reference = no use		0.825	0.000
Cancer stage: Reference = I	II	-0.126	0.383
	III	-0.085	0.574
	IV	-0.22	0.547
Systemic cancer treatment: Reference = none	Chemotherapy	0.55	0.008
	Radiotherapy	0.707	0.012
	Chemotherapy and radiotherapy	1.106	0.000
Age: Reference = young	Adult	1.731	0.085
	Older adult	2.196	0.030
Use of tamoxifen - Systemic cancer treatment: Reference = no use of tamoxifen and no treatment	Use of tamoxifen - chemotherapy	-1.011	0.000
	Use of tamoxifen - radiotherapy	-1.495	0.005
	Use of tamoxifen - chemotherapy and radiotherapy	-1.508	0.000
Constant		-1.676	0.102

Poisson regression analysis.

**Table 4:** Tooth loss in relation to the time of use of tamoxifen in patients with breast cancer adjusted for covariates.

Tooth loss		Coefficient	*p*
Time of use of tamoxifen: Reference = no use	Up to 1 year	0.623	0.003
	More than 1 year	0.964	0.000
Cancer stage: Reference = I	II	-0.126	0.385
	III	-0.083	0.588
	IV	-0.209	0.566
Systemic cancer treatment: Reference = none	Chemotherapy	0.549	0.008
	Radiotherapy	0.705	0.012
	Chemotherapy and radiotherapy	1.104	0.000
Age: Reference = young	Adult	1.671	0.097
	Older adult	2.144	0.034
Time of use of tamoxifen - Systemic cancer treatment: Reference = no use of tamoxifen and no treatment	Up to 1 year of use of tamoxifen - chemotherapy	-1.006	0.027
	Up to 1 year of use of tamoxifen - radiotherapy	-1.675	0.031
	Up to 1 year of use of tamoxifen - chemotherapy and radiotherapy	-2.369	0.022
	More than 1 year of use of tamoxifen - chemotherapy	-1.078	0.001
	More than 1 year of use of tamoxifen - radiotherapy	-1.255	0.055
	More than 1 year of use of tamoxifen - chemotherapy and radiotherapy	-1.52	0.000
	Constant	-1.618	0.115

Poisson regression analysis.

## References

[1] Araujo SF, Jardim LC, Ferrazzo KL, Skupien JA, Antoniazzi RP (2022). Association between tamoxifen and tooth loss in women with breast cancer. Support Care Cancer.

[2] Elani HW, Batista AFM, Thomson WM, Kawachi I, Chiavegatto Filho ADP (2021). Predictors of tooth loss: A machine learning approach. PLoS One.

[3] Mark AM (2020). Preventing tooth loss. J Am Dent Assoc.

[4] Pitchika V, Jordan RA, Norderyd O, Rolander B, Welk A, Völzke H (2022). Factors influencing tooth loss in European populations. J Clin Periodontol.

[5] Hag MS, Sabbah W (2023). Is tooth loss associated with multiple chronic conditions?. Acta Odontol Scand.

[6] Yin W, Wang J, Jiang L, James KY (2021). Cancer and stem cells. Exp Biol Med (Maywood).

[7] Diori KI, Sanlier SH (2021). Reviewing cancer's biology: an eclectic approach. J Egypt Natl Canc Inst.

[8] Youn HJ, Han W (2020). A Review of the epidemiology of breast cancer in Asia: Focus on Risk Factors. Asian Pac J Cancer Prev.

[9] Łukasiewicz S, Czeczelewski M, Forma A, Baj J, Sitarz R, Stanisławek A (2021). Breast Cancer-Epidemiology, risk factors, classification, prognostic markers, and current treatment Strategies-An Updated Review. Cancers (Basel).

[10] Ustaoğlu G, Göller Bulut D, Üyetürk Ü, Uysal Ö (2021). Evaluation of periodontal health in breast cancer patients undergoing tamoxifen or aromatase inhibitors drugs therapy: A cross-sectional study. Spec Care Dentist.

[11] Gadaleta E, Thorn GJ, Ross-Adams H, Jones LJ, Chelala C (2022). Field cancerization in breast cancer. J Pathol.

[12] Ferrillo M, Migliario M, Marotta N, Lippi L, Antonelli A, Calafiore D (2022). Oral health in breast cancer women with vitamin D deficiency: A Machine Learning Study. J Clin Med.

[13] Jardim LC, Flores PT, do Carmo Dos Santos Araújo M, Chiesa J, de Moraes CMB, Antoniazzi RP (2020). Oral health-related quality of life in breast cancer survivors. Support Care Cancer.

[14] Zhang Y, Ren X, Hu T, Cheng R, Bhowmick NA (2023). The relationship between periodontal disease and breast cancer: from basic mechanism to clinical management and prevention. Oral health prev dent.

[15] Wang T, Zhou Y, Cao G (2021). Pharmacogenetics of tamoxifen therapy in Asian populations: from genetic polymorphism to clinical outcomes. Eur J Clin Pharmacol.

[16] Ortiz J, Aranda FJ, Teruel JA, Ortiz A (2021). Dissimilar action of tamoxifen and 4-hydroxytamoxifen on phosphatidylcholine model membranes. Biophys Chem.

[17] Thorneloe RJ, Hall LH, Walter FM, Side L, Lloyd KE, Smith SG (2020). Knowledge of potential harms and benefits of tamoxifen among women considering breast cancer preventive therapy. Cancer Prev Res (Phila).

[18] Hosainzadegan H, Parvan R, Hosainzadegan M (2022). A retrospective study comparing oral health in cancer patients and healthy people. European journal of translational myology.

[19] Ali AH, Al Janabi (2021). Repurposing of tamoxifen against the oral bacteria. Turkish journal pharmaceutical Sci.

[20] Lucisano MP, da Silva RAB, de Sousa Pereira AP, Romualdo PC, Feres M, de Queiroz AM (2021). Alteration of the oral microbiota may be a responsible factor, along with estrogen deficiency, by the development of larger periapical lesions. Clin Oral Investig.

[21] Su X, Jin K, Zhou X, Zhang Z, Zhang C, Li Y (2023). The association between sex hormones and periodontitis among American adults: A cross-sectional study. Front Endocrinol (Lausanne).

[22] Shah R, Shah H, Thakkar K, Parikh N (2023). Conventional therapies of oral cancers: highlights on chemotherapeutic agents and radiotherapy, their adverse effects, and the cost burden of conventional therapies. Crit Rev Oncog.

